# A Structural Equation Modeling Analysis to Explore Diabetes Self-Care Factors in a Rural Sample

**DOI:** 10.3390/healthcare10081536

**Published:** 2022-08-14

**Authors:** Laurie Abbott, Lucinda Graven, Glenna Schluck, Jennifer Lemacks

**Affiliations:** 1 College of Nursing, Florida State University, Tallahassee, FL 32306, USA; 2 College of Nursing and Health Professions, University of Southern Mississippi, Hattiesburg, MS 39406, USA

**Keywords:** rural, diabetes, health promotion, disease risk, diabetes self-management

## Abstract

Diabetes is a public health problem that requires management to avoid health sequelae. Little is known about the determinants that influence diabetes self-care activities among rural populations. The purpose of this analysis was to explore the relationships among diabetes self-care activities, diabetes knowledge, perceived diabetes self-management, diabetes fatalism, and social support among an underserved rural group in the southern United States. A diabetes health promotion program was tested during a cluster randomized trial that tested a disease risk reduction program among adults living with prediabetes and diabetes. A structural equation model was fit to test psychosocial factors that influence diabetes self-care activities using the Information–Motivation–Behavioral Skills Model of Diabetes Self-Care (IMB-DSC) to guide the study. Perceived diabetes self-management significantly predicted self-care behaviors, and there was also a correlation between perceived diabetes self-management and diabetes fatalism. Perceived diabetes self-management influenced diabetes self-care activities in this rural sample and had an association with diabetes fatalism. The findings of this study can facilitate clinical care and community programs targeting diabetes and advance health equity among underserved rural groups.

## 1. Introduction

Chronic diseases such as heart disease, stroke, cancer, and diabetes are the leading causes of death, disability, and high health care costs [1,2]. In fact, approximately 6 out of 10 adults living in the United States have been diagnosed with at least one chronic disease, and 4 out of 10 have at least two chronic diseases [1,2]. Chronic diseases have been associated with risk behaviors including tobacco and alcohol use, physical inactivity, insufficient sleep, stress, and poor nutrition [2]. However, diabetes is an underrecognized contributory risk factor for chronic diseases such as cardiovascular disease, poor health outcomes, and increased mortality [3,4]. Diabetes is prevalent among rural populations in the southern United States, where life expectancy rates are the lowest in the nation, especially among African Americans who have the highest rates of chronic disease prevalence, morbidity, and mortality [4,5,6,7,8,9,10,11,12,13].

Populations living in rural areas bear a disproportionate chronic disease burden [7,8,10,14,15,16,17]. Compared with urban groups, those living in rural geographic locations have poorer health outcomes, fewer healthcare options, higher rates of premature death, and greater chronic disease risk factors [16,17,18]. Resource limitations, deficits in disease knowledge, and limited access to primary and preventive care further exacerbate disease risk [4,6,8,11,12,19,20]. In addition to these factors, worse outcomes among rural populations living with poorly controlled diabetes have been associated with their lack of adherence to disease management recommendations [21,22,23]. Poor glycemic control is associated with higher glycated hemoglobin (HbA1C) levels and contributes to the development and progression of chronic conditions such as heart disease and stroke [3,4].

Health outcomes associated with diabetes among rural, southern groups can potentially be improved through health promotion and disease risk reduction interventions that promote healthier lifestyle choices, improved knowledge about diseases and ways to reduce risk, and strengthen protective psychosocial factors [9,24,25]. However, there is a gap in knowledge regarding the determinants that influence diabetes self-care and management among diverse populations living in rural, southern states [22,26,27,28]. The Information–Motivation–Behavioral Skills Model of Diabetes Self-Care (IMB-DSC) was the theoretical framework used to guide this study, which indicates that performing diabetes self-care activities is influenced by psychosocial factors including diabetes knowledge, perceived diabetes self-management, diabetes fatalism, and social support [29]. Diabetes fatalism is described as the emotional distress from daily living with diabetes, coping using religious and spiritual resources, and self-efficacy in managing diabetes [30]. Social support is associated with improved diabetes self-management and outcomes [31,32,33,34]. More diabetes knowledge and social support and less diabetes fatalism increase the probability that diabetes self-care behaviors linked to glycemic control would be performed [29]. The purpose of this analysis was to explore the relationships among diabetes self-care activities, diabetes knowledge, perceived diabetes self-management, diabetes fatalism, and social support.

## 2. Materials and Methods

This study involved an analysis of data collected in a cluster randomized trial to test a culturally relevant disease risk reduction curriculum among people living with diabetes and prediabetes (ClinicalTrials.gov: NCT04795050). The study included 12 participating churches situated in a rural area of the southeastern United States, and the primary results showed statistically significant intervention effects for diabetes knowledge and some of the measured self-care activities [35]. The study received institutional review board approval at Florida State University.

### 2.1. Sample and Setting

The design of the cluster randomized trial involved calculating the number of participating churches and individual participants within churches needed to account for potential intra-cluster correlation [35]. For example, individuals within a participating church may share similar characteristics or kinship bonds that could have the effect of inducing correlation among study outcomes [36]. A conservative intra-correlation value (r = 0.008) from previous health research [37] and a medium standardized effect size (d = 0.50) from a similar study [38] were used to determine the sample size. For sufficient power (80%), the study required the inclusion of at least 5 different churches and at least 71 individual participants, accounting for 10% attrition, for each of the two groups. Serving as the statistical cluster, the rural churches situated in non-metropolitan areas as classified by ZIP codes were randomized to intervention and control groups after the pastors expressed interest in study participation. Randomization involved using a randomly selected five-digit random number sequence and assigning each church to either an intervention (even number) or control (odd number) group while ensuring the parity imbalance did not exceed two. The individual study participants were recruited from within the participating church congregations and self-identified as African American, were at least 22 years old, had previously been diagnosed with either diabetes or prediabetes, and could understand and speak English. Those deemed eligible and willing to participate provided written informed consent. The participants recruited from churches allocated to the intervention group received a culturally relevant diabetes risk reduction intervention, and those in churches randomized to the control group received a patient education sheet.

### 2.2. Measures

Participants in the intervention group completed the measures at baseline and post-intervention following the third weekly session while participants in the control group completed baseline measures and attended a second data collection period three weeks later. Sociodemographic information was collected at baseline, and the items included age, gender, employment status, educational attainment, previous diagnosis of either diabetes or prediabetes, diabetes management regimen, family history of heart disease or diabetes, and personal history of diabetic retinopathy. The self-reported measures for diabetes self-care activities, diabetes fatalism, perceived diabetes self-management, social support, and diabetes knowledge were collected during each data collection period.

#### 2.2.1. Diabetes Self-Care Activities

Diabetes self-care activities were measured using the Summary of Diabetes Self-care Activities, which had adequate internal consistency (α = 0.71) [39]. The instrument contains 15 items that measure self-care activities, such as blood sugar testing, dietary factors, medications, and foot care, using an 8-point Likert scale for the number of days (0–7) the behavior had been performed during the last week.

#### 2.2.2. Diabetes Fatalism

Diabetes fatalism was measured using the Diabetes Fatalism Scale, a 12-item, 6-point Likert scale that had good overall internal consistency (α = 0.80) [30]. The constructs of diabetes fatalism that were included in the instrument were the emotional distress (α = 0.86), religious and spiritual coping (α = 0.77), and perceived self-efficacy (α = 0.77) subscales. Greater diabetes fatalism was indicated by higher scores (Range 12–72) on the overall instrument, including these subscales.

#### 2.2.3. Perceived Diabetes Self-Management

Perceptions of diabetes self-management were measured using the Perceived Diabetes Self-Management Scale (PDSMS), which has eight questions and Likert scale-type answer options with total score possibilities ranging from 8 to 40 [40]. The instrument had good internal consistency (α = 0.83), with higher scores indicating a greater level of confidence in diabetes self-management.

#### 2.2.4. Social Support

The 20-item Medical Outcomes Study Social Support Survey was used to measure social support in this study [41]. The instrument had 1 fill-in-the-blank item to record the number of close friends and relatives and 19 Likert-scale, 5-point options. The total social support score as well as the subscales (tangible support, emotional/informational support, affectionate support, and positive social interaction) had excellent internal consistency (α = 0.91–0.97). The tangible support items measured help provided by others during an illness such as providing transportation to a doctor and assisting with daily chores. Emotional/informational support measured the availability of someone else to share concerns, assist during a crisis, and provide advice. Affectionate support can be described as having someone to show displays of love and affection, and positive social interaction involves having someone to share enjoyable things with.

#### 2.2.5. Diabetes Knowledge

The Revised Diabetes Knowledge Test had adequate reliability (α = 0.77) and was used to measure diabetes knowledge [42]. The measure includes 23 multiple choice questions, but 3 items about insulin were not scored because the curriculum did not address insulin therapy. The 20 items were totaled, for a maximum possible score of 100 points.

### 2.3. Intervention

The participants in the intervention group received a manualized culturally relevant curriculum that was developed by the American Diabetes Association to improve diabetes self-management and reduce disease risk among African American adults in church settings. The program was delivered by the same advanced public health nurse in the participating churches randomized to the intervention group over 3 weekly sessions that lasted approximately 90 minutes to 2 hours, depending on questions and discussion. The sessions were similar to other diabetes health promotion interventions and included information about diabetes risk and pathology, managing diabetes and prediabetes through diet and exercise, maintaining glucose and HbA1C levels within therapeutic ranges, and linkages with heart disease, kidney damage, and stroke. Interactive strategies were employed, which facilitated group discussion and engagement. The control group received a brochure about diabetes.

### 2.4. Data Analysis

A confirmatory factor analysis (CFA) model was fit to test the measurement model where self-care behavior, a latent variable, was predicted to load onto eight measured variables. These measured variables are general diet, specific diet related to fat intake, specific diet related to carbohydrate intake, specific diet related to produce intake, exercise, blood glucose testing, foot care, and medication management. The CFA model was nested into the full structural model testing the hypothesis that diabetes knowledge, diabetes fatalism, perceived diabetes self-management, and social support predict self-care behaviors. The hypothesized model is similar to the one proposed in a previous publication by Osborn and Egede, although the specific diet subscale is three individual subscales, and there is no smoking subscale included [29]. Additionally, perceived diabetes self-management was included as a predictor of self-care behaviors. The structural model and the CFA model were tested using MPlus version 8.5 [43]. Variables with factor loadings that were not significant were excluded from the full SEM analysis. Model fit was assessed with likelihood ratio chi-square tests and the root mean square error of approximation (RMSEA) [44,45]. Insignificant chi-square test results indicate that the data fit the hypothesized structure. RMSEA values less than 0.05 indicate a close fit, whereas values between 0.05 and 0.08 indicate a reasonable fit. RMSEA values larger than 0.1 indicate a poor fit [46].

## 3. Results

The sample included 12 participating churches that were randomized to the intervention (*n* = 7) and control (*n* = 5) groups. Of the 146 individual participants recruited from the randomized churches, 75 received the intervention, and 71 were in the waitlisted control group. However, a few participants (*n* = 9) from both the intervention (*n* = 7) and control (*n* = 2) groups ceased participation after providing informed consent and completing baseline measures [35]. All self-identified as African American, and there were no significant between-group differences regarding sociodemographic characteristics except that more participants in the intervention group had previously been diagnosed with diabetic retinopathy (*p* = 0.003) and were unemployed (*p* = 0.02) [35]. The average ages for participants in the intervention group were 61.8 and 61.6 for those in the control group, and there were more participants (72%) who self-identified as being unemployed in the intervention group, compared with those (49%) in the control group [35]. Additionally, women (*n* = 110; 75%) participated in the study more than men (*n* = 36; 25%), but there was no statistically significant gender difference (*p* = 0.08) between groups [35]. Similar numbers of people in both the intervention (*n* = 43) and control (*n* = 44) groups had been diagnosed with diabetes, and of those diagnosed with prediabetes, there were a few more in the intervention group (*n* = 32), compared with the control group (*n* = 27). 

**Figure 1:**
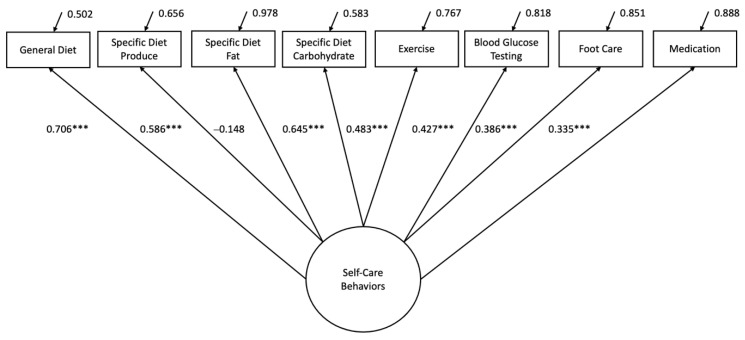
Confirmatory factor analysis (CFA) model. Standardized CFA solution; *** *p* < 0.001.

The CFA model is graphically depicted in Figure 1. The initial CFA model demonstrated a poor fit (χ^2^ (20) = 54.450, *p* < 0.001; RMSEA = 0.109) to the data; thus, model adjustments were made. First, the measured variable for specific diet related to fat intake was removed, as it was insignificant. This model also demonstrated a poor fit to the data (χ^2^ (14) = 48.256, *p* < 0.001; RMSEA = 0.129), and therefore, a further refinement was made by adding the correlation between blood glucose testing and medication management to the model. The adjusted model demonstrated an adequate fit (χ^2^ (13) =13.224, *p* = 0.4307; RMSEA = 0.011 [90%CI: 0.000, 0.083]). Diabetes self-care behaviors loaded significantly onto general diet (0.719, *p* < 0.001), specific diet for produce intake (0.592, *p* < 0.001), specific diet for carbohydrate intake (0.643, *p* < 0.001), exercise (0.511, *p* < 0.001), blood glucose testing (0.372, *p* < 0.001), foot care (0.374, *p* < 0.001), and medication management (0.258, *p* = 0.006). 

**Figure 2:**
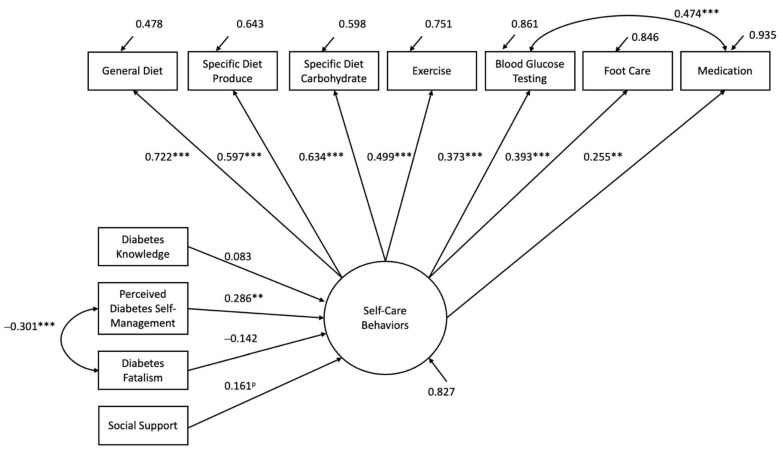
Structural model with parameter estimates for each path. Standardized solution: ** *p* < 0.01, *** *p* < 0.001, p indicates a *p*-value equal to 0.085. Only one correlation among exogenous variables is presented, although all were fit. See Table 1 for correlations.

The structural model that was estimated is displayed with parameter estimates for each path in Figure 2. The estimated model showed a goodness of fit (χ^2^ (37) = 31.906, *p* = 0.7065; RMSEA = 0.000 [90%CI: 0.000, 0.046]). The resulting model had no multivariate outliers or issues with collinearity. The path beginning with perceived diabetes self-management was the only significant direct path predicting self-care behaviors (r = 0.286, *p* = 0.002). 

Additionally, the correlation between perceived diabetes self-management and diabetes fatalism was also significant (Table 1). Model results indicated that higher perceived diabetes self-management, higher diabetes knowledge, less diabetes fatalism, and more social support predicted better self-care behaviors, with the model explaining 17.3% of the variability in diabetes self-care behaviors.

## 4. Discussion

The IMB-DSC helped conceptualize the determinants of diabetes self-care activities among rural participants living with diabetes and prediabetes including diabetes knowledge as information, diabetes fatalism as personal motivation, social support as social motivation, and diabetes self-care as health behaviors [29]. The current study included the same components with the addition of the perceived diabetes self-management scale to represent self-efficacy in relation to diabetes self-care activities [40]. As a chronic disease, diabetes requires individual confidence, or self-efficacy, to perform the diabetes self-management activities necessary for preventing adverse consequences associated with lack of glycemic control [40]. Little is known about factors that influence diabetes self-care outcomes, and the findings of this study contribute to the literature about diabetes outcomes among underserved rural groups. In this sample, the intervention had no statistically significant impact on perceived diabetes self-management, and a possible rationale could be that the levels were already high at baseline and left little room for improvement at post-test [47]. However, in this analysis, perceived diabetes self-management had the largest impact on self-care behaviors, as greater self-efficacy in performing self-care activities was associated with the actual performance of those behaviors. Similarly, another study conducted among Chinese participants concluded that self-efficacy was an independent predictor of diabetes self-care behaviors [48]. 

There are a few published studies that discussed perceived diabetes self-management in other populations. For example, a study that included adults in Saudi Arabia showed a significant correlation between perceived diabetes self-management and social support [49]. Another showed that a telephone intervention delivered to Turkish adults significantly improved diabetes self-efficacy and perceived self-management [50]. Additionally, motivational interviewing had a significant impact on perceived diabetes self-management in Turkish adults [51]. An educational health technology program discussed in another study improved the perceptions of diabetes self-care as well as glycemic control among study participants in Asia [52]. The positive associations and promising intervention delivery methods discussed in these articles provide important considerations when developing future public health interventions targeting diabetes.

In this sample, the negative correlational relationship between perceived diabetes self-management and diabetes fatalism was statistically significant. This result makes clinical sense since, with an increase in the perception of diabetes self-management, it would seem likely that people would have greater feelings of self-efficacy and fewer fatalistic beliefs about living with and managing diabetes. However, even though the perceived diabetes self-management score was reported as high in this population, diabetes fatalism was moderate, which suggests that interventions should include efforts to reduce fatalism by directly impacting psychological and behavioral factors [47,53]. This has clinical importance because other studies found that higher diabetes fatalism was directly linked to poor glycemic control, poor medication adherence, and decreased self-care [53,54,55]. The results of this study showed that diabetes fatalism had a small negative effect in that lower levels of fatalism were associated with a greater likelihood of performing diabetes self-care behaviors. In comparison, having more social support and increased diabetes knowledge had small positive effects on self-care activities. A future study having a larger sample size could potentially show larger effects on these outcomes in this population. 

Future research can also focus on the impact of individual perceptions of diabetes self-management on self-care behaviors in similar rural groups as well as urban populations. The strategies previously discussed such as using motivational interviewing, health technology, and telephone interventions could be used to enhance diabetes health interventions and improve accessibility among underserved populations residing in any location. Poverty and poor living conditions and built environment conditions can adversely affect diabetes outcomes [56]. Further research is needed to explore the impact of social determinants of health on factors related to diabetes self-care, such as fatalism, self-efficacy, and social support in other geographic areas, and brainstorm strategies to improve health among diverse populations living with diabetes. Additionally, a future analysis can explore the impact of sociodemographic characteristics and diabetes-related complications on outcomes. For example, a study among Lebanese adults showed that fatalistic attitudes were associated with characteristics such as younger age, lower educational attainment, higher BMI, and fewer diabetes comorbidities [57]. This study had some limitations as well. First, the study was conducted among participants living in a southern region of the United States, which could limit generalizability to other populations. Second, the data were self-reported by participants, and there could have potentially been recall bias when completing the survey instruments.

## 5. Conclusions

Confronting inequalities requires solutions that are practical, relevant, and encompass the unique characteristics of those communities that have experienced multiple geographic and systemic disadvantages [58]. For example, people living in rural geographic locations often experience hardships related to poverty and limited access to health information, access to health care, and other resources that affect health program responses and impact outcomes. The COVID-19 pandemic has further exacerbated these challenges and will likely contribute to a resurgence of diabetes-related sequelae in addition to the consequences of other chronic diseases. Mitigating rural disparities associated with diabetes involves community-relevant strategies that address the psychological and behavioral determinants of standardized diabetes care that improve diabetes self-care activities [53] Healthy lifestyle recommendations promote longer life expectancy and reduce chronic disease risk associated with the major causes of death associated with diabetes such as cancer, stroke, and cardiovascular disease [59,60,61]. The development and testing of such tailored interventions can potentially impact health outcomes, decrease chronic disease development and exacerbation, and improve the quality of life among rural dwellers.

## Figures and Tables

**Table 1:** Correlations.

	Diabetes Knowledge	Perceived Diabetes Self-Management	Diabetes Fatalism	Social Support
Diabetes Knowledge	1	−0.098	−0.066	0.155
Perceived Diabetes Self-Management		1	−0.301 ***	0.101
Diabetes Fatalism			1	−0.074

Note. ***p < 0.001.

## Data Availability

The data presented in this study will be considered upon request from the corresponding author. The data are not publicly available due to privacy issues.
